# Burden of prostate cancer and relationship with the human development index (HDI) in Asia: A study of Global Burden disease in 2019

**DOI:** 10.22088/cjim.14.4.710

**Published:** 2023

**Authors:** Fazlollah Fathollahi, Zaher Khazaei, Mahshid Abbasi, Elham Goodarzi

**Affiliations:** 1 Assistant Professor Nutritional Health Research Center, school of Medicine, Lorestan University of Medical Sciences, khoramabad, Iran; 2 Center for Healthcare Data Modeling, Departments of biostatistics and Epidemiology, School of public health, Shahid Sadoughi University of Medical Sciences, Yazd, Iran.; 3 Assistant Professor School of Medicine Shahid Rahimi Hospital Lorestan University of Medical Sciences khoramabad, Iran; 4 Social Determinants of Health Research Center, Lorestan University of Medical Sciences, Khorramabad, Iran

**Keywords:** Prostate cancer, Inequality, Incidence, Mortality, Disease burden.

## Abstract

**Background:**

Prostate cancer (PC) ranks as the second most commonly diagnosed neoplasia and the fifth cause of death in men with cancer, with an increasing trend in incidence.

**Methods:**

All accessible data sources from the 2019 Global Burden of Disease study were used to estimate the prevalence, mortality and disability-adjusted life years (DALY) and burden prostate cancer in Asia from 1990 to 2019. We estimated all-cause and cause-specific mortality, years of life lost (YLLs), years lived with disability (YLDs) and DALYs. All estimates were presented as counts and age-standardized rates per 100 000 population, with uncertainty intervals (UIs). Concentration Index analysis and Concentration Curve were used to determine the relationship between Prostate cancer burden and human development index.

**Results:**

The results showed that the percentage of changes in the incidence in 1990-2019 was positive in all countries of the Asian continent except for Afghanistan and Kyrgyzstan. The results of the concentration index showed that the incidence and mortality of prostate cancer is more concentrated in countries with a high HDI level. Examining the DALY, YLL and YLD index also showed the value of concentration index, which shows that DALY, YLL and YLD of prostate cancer are more concentrated in countries with high HDI level.

**Conclusion:**

Given that burden of prostate cancer are increasing in most Asian countries and are mostly concentrated in the HDI drawers, obtaining accurate estimates in these countries to prepare for the potential change in public health burden due to this disease which is very important.

**T**oday, non-communicable diseases (NCDs) are the main cause of death in the world. According to WHO estimates in 2015, cancer is the first or second cause of death before the age of 70 in most countries of the world. One of the reasons for the increase in the prevalence of cancer in the whole world is population growth, increase in life expectancy and economic development (1). Prostate cancer is one of the important cancers whose prevalence has increased in recent years. Prostate cancer is the fifth cause of death worldwide and the second most common cancer after lung cancer in men (2, 3). Prostate and its impact on patients' quality of life, economic costs and mortality are important and significant cancers (4). One of the most important factors affecting the occurrence of prostate cancer is aging, obesity, inactivity, smoking, and a high-fat diet (5). Environmental and economic-social factors are also influential in prostate cancer mortality. The general trend of prostate cancer incidence and death can be observed in all regions of the world and in all levels of economic-social development (6).

The only difference in the incidence of prostate cancer in the world is not only due to the factors that cause it, but it can also be due to the availability of primary screening and the accurate registration system of the disease in developed countries (7).

Globally, there is a great variation in the rate of diagnosis of new cases of prostate cancer. The continuation of the global demographic and epidemiological trend is a clear indicator of the increase of cancer during the coming decades, especially in low and middle income countries (8). The prevalence of prostate cancer is higher in the countries of South and East Asia, Europe and North America (9). Also, this rate is higher in the developed countries than in the developing countries (10). The highest incidence rate of this cancer in 2016 was in Australia and then in the United States, and the lowest incidence rate was seen in African countries (11). According to the latest report from the National Vital Statistics System, the death rate is higher in the elderly and in whites in relation to other races (10, 12). The death rate has increased in people over 85 years old (7). The incidence of prostate cancer in men in developed countries is higher than in developing countries (2, 8, 13). While the highest age-specific death rate of prostate cancer is related to developing countries. Also, in relation to the Human Development Index (HDI), in areas with high HDI, the burden of diseases caused by prostate cancer is higher (1). Previous research has identified a strong correlation between human development or socioeconomic progress and cancer (14-16). 

Current studies provide valuable evidence for decision-making and setting priorities for addressing the burden of cancer, but also raise questions about disparities in incidence, mortality, and burden. In this review, we summarize the links between prostate cancer burden and human development indicators and highlight disparities in burden across levels of human development. Looking at how human development levels affect cancer profiles, the most common cancers in high and very high HDI countries are prostate, breast, colorectal and lung cancers (14).

In this study, we intend to investigate the key characteristics of prostate cancer transmission in the Asian continent by examining the relationship between the incidence rate and the human development index, which consists of life expectancy, education, and gross national income. 

## Methods

This study is a correlational analysis study whose purpose was to investigate the relationship between prostate cancer epidemiology and the human development index in Asia in 2019. The burden of diseases study is the most comprehensive and accurate global epidemiological study. The burden of diseases study is the result of examining 369 diseases and injuries and 87 health risk factors in 204 countries and regions of the world. Information related to this study, including disease burden index, years of life lost due to premature death and years of life lost due to disability were extracted from prostate cancer patients on the Global Burden of Disease website (17).

**Disability-Adjusted Life Years (DALY): **DALY is a type of health gap index that calculates the years of life lost, either due to premature death or due to non-fatal diseases. This index was defined and used in the Global Burden of Diseases and Injuries (GBD) study to calculate the burden of diseases (18).

**Years of life lost due to premature death (Years of Life Lost, YLL): **To identify and prioritize the causes of premature deaths, it is possible to use the index of years of life lost due to premature death (YLL), which was introduced by the World Health Organization in the study of the global burden of diseases. This index depends not only on the number of deaths but also on the age of the deceased at the time of death, and the younger the age of the deceased at the time of death, the more the number of lost years of life increases. The lost years of life are the years that a person could have had a useful life, but due to premature death, these years have been lost (18).

**Years Lived with Disability (YLD**): It has been years that a person has been disabled or disabled due to illness (18).

**Human Development Index (HDI):** The human development index for all developing and developed countries is estimated annually and is placed on the World Health Organization website for free use by researchers, and in this research, the information related to this index is also extracted from the World Health Organization website.The Human Development Index provided by the World Health Organization provides the most recent information on global development and includes national, regional and global estimates. In the human development report, countries are divided into groups of countries with very high human development, countries with high human development, countries with medium human development and countries with low (low) human development based on the human development index. The numerical value of human development index is between zero and one. The value of the human development index shows how much each country has traveled to reach the highest possible value, i.e., one, and also enables comparison between countries. Human Development Index (HDI) is a summary of human development measurements. This index measures the average success achieved in a country in the three main dimensions of human development, i.e. long and healthy life, knowledge acquisition and living standards (19, 20).

**Concentration Index: **The concentration index has desirable characteristics, such as the correctness of measuring the inequality related to income in the distribution of the health variable, and it can be easily calculated, and it quantitatively shows the degree of inequality in the level of the income distribution of a health variable, and with reference is defined to the concentration curve. The concentration curve (Lorenz Curve) on the X axis shows the cumulative percentage that are ranked based on income or socioeconomic status (in this study based on the HDI index) and on the y axis the cumulative percentage of the health variable (in this study incidence, mortality and shows disease burden) (21, 22).

The Lorenz curve is drawn as graph number one and is the basis of Gini coefficient and concentration index calculations. If the curve is below the diagonal line, it indicates the concentration of the health status variable (incidence, mortality and disease burden) in countries with high HDI, and if it is above the diagonal line, it indicates the concentration of the health status variable (incidence, mortality and disease burden) disease) is in the category of countries with low HDI. Tangency of the Lorenz curve with the diagonal line indicates the absence of inequality. 

The amount of inequality will be equal to twice the area between the curve and the diameter. Concentration index values ​​vary between +1 and -1 and it is one of the common indicators in calculating inequalities related to income or socioeconomic status. Its negative values ​​indicate that the health variable (incidence, mortality and disease burden) is concentrated between countries with low HDI and the concentration curve is above the equality line, but positive values ​​indicate that the health variable (incidence, mortality) and disease burden) is concentrated among countries with high HDI and the concentration curve lies below the equality line. When the distribution of the health variable (incidence, mortality and disease burden) is the same among all countries, the concentration index will be equal to zero (22).

**Data analysis:** In this study, bivariate correlation method was used to analyze the extracted data to check the correlation between prostate cancer burden and HDI. The significance level was p>0.05. Analyzes were performed using Stata software Version 12 (Stata Corp, College Station, TX, USA).

## Results

**Figure 1:**
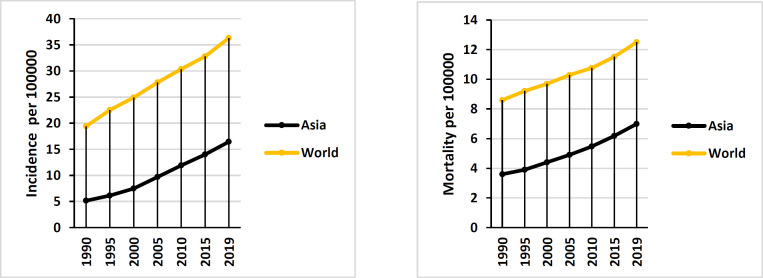
Incidence and mortality rate of prostate cancer in the world and Asia during 1990-2019 (source: Global Burden of Disease)

**Figure 2:**
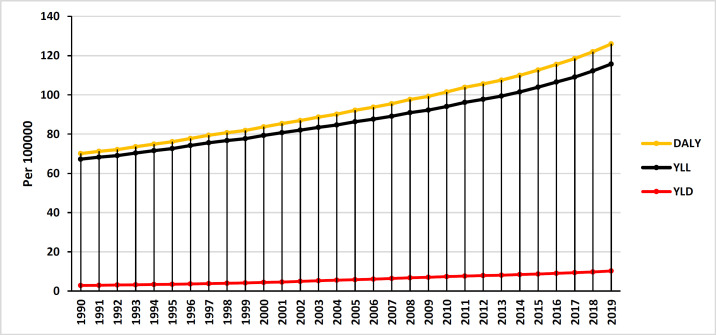
DALY, YLL and YLD trend of prostate cancer in Asia during 1990-2019

Figure 1 shows the trend of prostate cancer incidence and mortality during the years 1990 to 2019 in the world and the Asian continent and mortality (8.6 per 100,000 in 1990 to 12.5 per 100,000) of prostate cancer has increased. Also, in the Asian continent, the trend of incidence (from 5.15 per 100,000 in 1990 to 16.44 per 100,000) and mortality (from 3.6 per 100,000 in 1990 to 6.99 per 100,000 in 2019) has been increasing. The results show that the amount of DALY, YLL and YLD of prostate cancer in the Asian continent has increased during the period of 1990 to 2019 (figure 2).

**(Source: Global Burden of Disease)**

According to the results presented in table 1, the highest incidence of prostate cancer in the Asian continent in 2019 is related to the countries of Japan (5.90 per 100,000), Lebanon (61.67 per 100,000) and Israel (31.48 per 100,000) and the highest mortality rate is related to the countries of Georgia (27.87 per 100,000), Japan (26.35 per 100,000) and Lebanon (23.08 per 100,000). The results show that the highest rate of DALY, YLL and YLD of prostate cancer is related to Georgia. The results showed that the percentage of changes in the incidence in 1990-2019 was positive in all countries of the Asian continent except for Afghanistan and Kyrgyzstan. And the changes in the mortality rate of the heads of the provinces during the years 1990-2019 have been positive in all Asian countries except Afghanistan, Israel, Kyrgyzstan, Pakistan, Saudi Arabia and Tajikistan (table 1). 

**Table 1:** Prostate cancer incidence, mortality and burden in 2019 in Asian countries (source: Global Burden of Disease)

**Per 100000**			**Percentage change in mortality rate**	**Mortality per 100000**	**Percentage change in incidence**	**Incidence per 100000**	**Country**
**DALY**	**YLD**	**YLL**	**1990-2019**		**1990–2019**		
47.83	1.36	46.46	-0.59(-0.68, -0.47)	2.57	-0.53(-0.65, -0.39)	2.98	**Afghanistan**
365.84	22.94	342.89	2.33(1.15, 3.2)	19.54	3.43(1.88, 4.66)	38.40	**Armenia**
171.73	8.46	163.27	0.5(0.16, 0.96)	7.88	1.07(0.59, 1.66)	14.08	**Azerbaijan**
92.94	11.51	81.44	0.39(-0.01, 0.94)	4.05	2.53(1.32, 4.33)	18.48	**Bahrain**
94.90	3.27	91.63	0.86(0.34, 1.43)	5.64	1.35(0.69, 2.17)	6.97	**Bangladesh**
98.00	3.60	94.40	2.72(1.62, 4.04)	5.91	3.60(2.21,5.33)	7.50	**Bhutan**
116.18	8.48	107.70	0.89(0.38, 1.55)	5.82	1.80(1.05, 2.84)	13.17	**Brunei**
151.76	5.66	146.10	1.13(0.65, 1.71)	8.13	1.79(1.11, 2.55)	10.93	**Cambodia**
138.32	39.04	124.68	1.25(0.72, 1.95)	7.50	3.89(2.64, 5.79)	21.17	**China**
560.34	119.39	480.72	1.63(0.83, 2.33)	27.87	0.57(0.05, 1.51)	46.19	**Georgia**
81.34	2.88	78.46	0.84(0.43, 1.5)	4.50	1.25(0.75, 2.02)	5.83	**India**
164.94	6.52	158.42	1.52(0.91, 2.29)	8.71	2.21(1.42, 3.26)	12.32	**Indonesia**
158.18	14.49	143.69	1.98(1.44, 2.74)	9.15	3.10(2.25,4.26)	24.34	**Iran**
74.91	5.10	69.81	0.34(-0.02, 0.85)	3.99	1.23(0.57, 2.23)	8.89	**Iraq**
261.07	32.09	228.97	-0.04(-0.2, 0.27)	16.49	0.65(0.16, 1.42)	48.31	**Israel**
393.57	63.31	330.26	1.79(1.35, 2.01)	26.35	3.16(2.28, 4.18)	90.50	**Japan**
78.07	7.22	70.85	0.76(0.32, 1.35)	4.13	2.19(1.3, 3.4)	11.89	**Jordan**
146.85	9.18	137.67	0.37(0.16, 0.66)	6.75	1.02(0.63, 1.46)	14.59	**Kazakhstan**
81.25	11.11	70.14	1.77(1.22, 2.42)	4.46	2.43(1.57, 3.47)	18.09	**Kuwait**
67.38	3.26	64.12	-0.19(-0.35, 0.24)	3.49	-0.01(-0.24, 0.68)	5.62	**Kyrgyzstan**
109.15	3.38	105.77	0.38(0.06, 0.78)	5.81	0.65(0.26, 1.14)	7.02	**Lao People's Democratic Republic**
384.88	41.19	343.69	1.09(0.46, 1.95)	23.08	2.8(1.64, 4.38)	67.61	**Lebanon**
130.07	8.96	121.11	0.89(0.44, 1.6)	7.12	2.12(1.33, 3.3)	14.74	**Malaysia**
56.72	4.33	52.39	0.53(0.14, 1.02)	3.39	1.74(0.92, 2.74)	7.34	**Maldives**
68.00	2.70	65.30	0.21(-0.09, 0.62)	3.14	0.65(0.22, 1.22)	4.67	**Mongolia**
152.39	5.45	146.93	0.87(0.47, 1.46)	8.30	1.33(0.79, 2.07)	10.75	**Myanmar**
88.98	2.82	86.16	1.31(0.65, 2.23)	5.09	1.78(0.94, 2.82)	6.00	**Nepal**
41.61	5.43	36.18	0.01(-0.28, 0.54)	1.83	1.26(0.58, 2.19)	8.33	**Oman**
83.51	2.44	81.07	-0.11(-0.37, 0.36)	4.29	0.11(-0.26, 0.73)	5.20	**Pakistan**
146.25	6.24	140.01	0.34(-0.04, 0.96)	7.48	0.63(0.13, 1.47)	11.25	**Philippines**
39.65	6.70	32.95	0.25(-0.13, 0.83)	1.48	2.72(1.35, 4.71)	10.49	**Qatar**
54.43	7.03	47.40	-0.09(-0.38, 0.88)	2.30	1.9(0.84, 5.04)	11.04	**Saudi Arabia**
158.24	23.34	134.91	0.99(0.44, 1.26)	8.65	2.98(1.84, 4.31)	34.11	**Singapore**
117.00	9.13	107.87	0.67(0.22, 1.26)	6.21	2.06(1.2, 3.25)	14.97	**Sri Lanka**
123.39	10.17	113.22	1.17(0.48, 2.45)	6.40	2.83(1.5, 5.11)	16.55	**Syrian Arab Republic**
58.65	2.08	56.57	-0.07(-0.31, 0.34)	2.83	0.08(-0.17, 0.54)	3.88	**Tajikistan**
182.83	13.61	169.22	1.61(0.91, 2.55)	10.43	3.16(1.91, 4.9)	22.83	**Thailand**
126.06	4.16	121.90	2.48(1.44, 4.07)	6.92	3.16(1.9, 5.18)	8.57	**Timor-Leste**
126.06	20.65	197.01	0.82(0.34, 1.42)	12.62	2.6(1.57, 3.96)	34.06	**Turkey**
92.02	4.25	87.77	0.54(0.23, 0.95)	4.01	1.09(0.64, 1.67)	7.01	**Turkmenistan**
59.61	6.14	53.47	0.57(0.04, 1.28)	1.89	2.47(1.15, 4.26)	9.30	**United Arab Emirates**
54.23	2.76	51.46	0.05(-0.13, 0.73)	2.44	0.51(0.2, 1.48)	4.42	**Uzbekistan**
114.74	7.37	107.37	0.82(0.37, 1.52)	6.09	2.1(1.27, 3.38)	12.17	**Viet Nam**
63.93	2.67	61.26	0.76(0.31, 1.53)	3.38	1.05(0.5, 2.04)	4.97	**Yemen**
180.90	27.05	153.85	2.56(1.25, 3.22)	10.05	7.62(4.39, 9.89)	39.32	**Republic of Korea**
130.96	7.01	123.95	1.04(0.61, 1.64)	6.54	1.18(0.66, 1.88)	11.58	**Democratic People's Republic of Korea**

**Figure 3:**
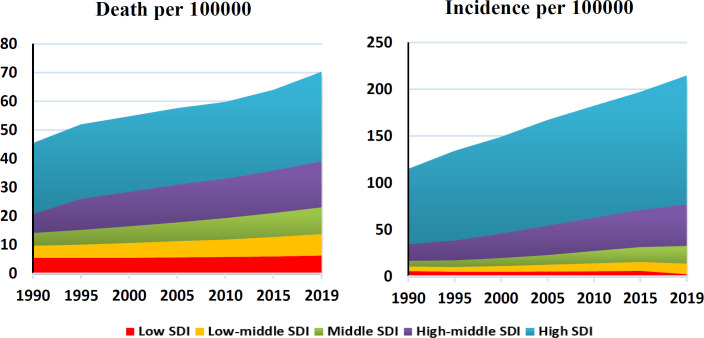
Incidence and death of prostate cancer in the world based on SDI level in 1990-2019

**Figure 4:**
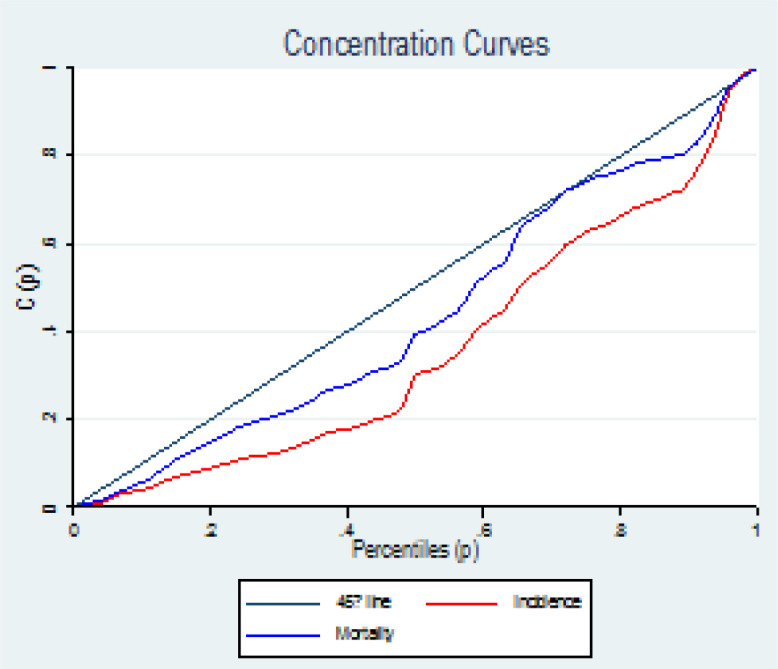
Prostate cancer incidence and mortality concentration index curve in Asia

Figure 3 shows the rate of incidence and death from prostate cancer in the development levels in the world during 1990-2019. As can be seen, the trend of incidence and mortality in all levels of development is an increasing trend, and the highest incidence and death from prostate cancer are related to high SDI and the lowest corresponds to low SDI (figure 3). Figure 3 shows the rate of incidence and death from prostate cancer in the development levels in the world during 1990-2019. As can be seen, the trend of incidence and mortality in all levels of development is an increasing trend, and the highest incidence and death from prostate cancer are related to high SDI and the lowest corresponds to low SDI (figure 3). In the examination of the index of concentration in the incidence and mortality of prostate cancer, the results showed that there is an inequality in the incidence and mortality of prostate cancer in Asian countries, and the incidence and mortality of this cancer is more concentrated in countries with a high HDI level, and the curve is located below the equality line (figure 4). 

**(Source:** **Global Burden of Disease** **)**

**Figure 5:**
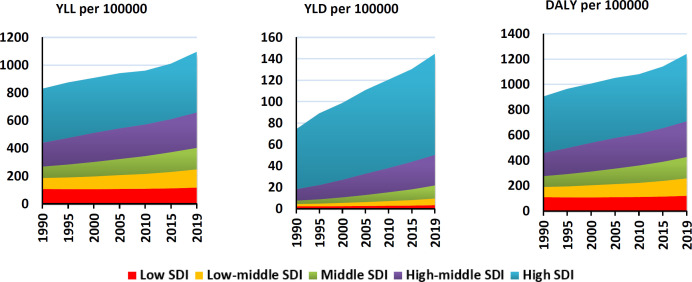
**Burden of prostate cancer in the world based on SDI level** ^*^ **in 1990-2019 (source: Global Burden of Disease)**

**Figure 6:**
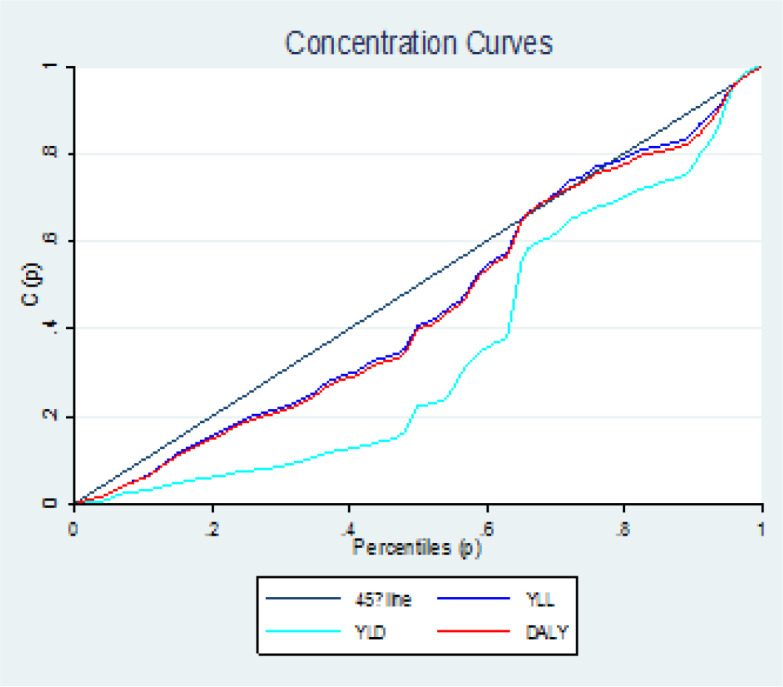
**Concentration index curves for DALY, YLL and YLD of prostate cancer** **.**

Table 2 shows the concentration index of prostate cancer incidence and mortality in the human development index composites. As can be seen in all the human development index composites, the prostate cancer concentration index is positive, which shows the incidence and mortality of prostate cancer in countries with a high human development index (table 2). Figure 5 shows the trend of DALY, YLL and YLD during the years 1990 to 2019 based on development. As can be seen, the highest DALY, YLL and YLD from prostate cancer are related to high SDI areas and the lowest are related to low SDI areas.is (figure 5). In examining the DALY, YLL and YLD index, it can be seen that the DALY, YLL and YLD curves are below the equality line, which shows that prostate cancer is more concentrated in countries with a high HDI level (figure 6). 

**Table 2:** Prostate cancer incidence and mortality concentration index based on human development index composites

**Mortality**	**Incidence**	
0.12(0.009, 0.23)	0.27(0.14, 0.41)	**HDI**
0.15(0.03, 0.27)	0.32(0.19, 0.45)	**Life expectancy at birth**
0.13(0.01, 0.26)	0.24(0.09, 0.4)	**Mean year of schooling**
0.15(0.01, 0.29)	0.23(0.08, 0.38)	**Expected years of schooling**
0.05(-0.06, 0.16)	0.19(0.06, 0.33)	**Gross national income per 1000 capita**

*SDI (Socio-demographic Index): A summary measure that identifies where countries or other geographic areas sit on the spectrum of development. Expressed on a scale of 0 to 1, SDI is a composite average of the rankings of income per capita, average educational attainment, and fertility rates of all areas in the GBD study (23, 24).

## Discussion

The results of this research showed that the highest incidence of prostate cancer in the Asian region was related to the countries (90.5 per 100,000), Lebanon (67.61 per 100,000) and Israel (48.31 per 100,000). The highest prostate cancer death rates were in Georgia (27.87 per 100,000), Japan (26.35 per 100,000) and Lebanon (23.08 per 100,000). The highest rate of DALY, YLL and YLD of prostate cancer was in Georgia. The percentage of incidence changes in the years 1990-2019 was positive in all countries of the Asian continent except for Afghanistan and Kyrgyzstan. And the changes in the mortality rate of prostate cancer during the years 1990-2019 in all Asian countries except Afghanistan, Israel, Kyrgyzstan, Pakistan, Saudi Arabia and Tajikistan have had an upward trend. The difference in the incidence of cancer in these regions can be due to the difference in economic status. It is also social of people.

In countries with high levels of HDI, the incidence and mortality rate of prostate cancer is higher. Among the reasons for the high incidence, we can point to things like the existence of a screening program, high level of health care, rapid treatment of the disease in the early stages, and the accurate registration system in these countries (11, 25).

More than 50% of prostate cancer deaths occurred in developed countries (1, 4). Incidence and death from this cancer have decreased in the United States. But it is increasing in some European countries and Asian developing countries (26). 

The highest incidence rate of prostate cancer is in Australia, New Zealand with 84.6 per hundred thousand people and North and Western Europe, and the incidence rate is observed in South Central and Southeast Asia at 5 per hundred thousand people (16). Countries with a low level of GDP such as Tajikistan, Turkmenistan, Uzbekistan, Bangladesh, Nepal and Greece have the lowest incidence of prostate cancer (5).The results of the study by Chuan Wang et al. in 2020 showed that the incidence rate of this cancer is different in different races. In the United States, the highest incidence rate is among African-American men and the lowest incidence rate is among Native Americans and Alaskans.These countries had medium and high HDI.In this study, a significant positive correlation was seen between HDI (income level) and the incidence of prostate cancer (27). The results of the study by Seraphin1 et al. in 2021 showed that the incidence of prostate cancer in the South African desert will triple by 2040.This indicates that there is an inverse relationship between the level of HDI (education, income) and mortality (28).

Sharma et al.'s study in 2019 showed that there is a significant positive relationship between HDI levels and the incidence of prostate cancer (p<0.001). So, the incidence of prostate cancer is higher in high-income countries. The findings of this study were consistent with the results of research (13). These studies show that cancer is still a global and important issue in health societies and there is a need for more extensive studies to understand the relationship between economic development and prostate cancer. Social and economic developments have had a significant impact on the incidence and attenuation of cancer. In countries with low or middle income, the risk of cancers is increasing. Cancer-causing infections account for about 26% of cancers in middle- and low-income countries. Increase in life expectancy, aging, lifestyle change and exposure to biological hazards are known as prostate cancer risk factors (29). Among the reasons for the high incidence of prostate cancer in countries with a higher HDI, we can mention the existence of suitable infrastructure, advanced diagnostic methods, good access to basic health services, high standard of living and early screening. Also, the reason for the higher mortality in developing countries are the lack of access to proper diagnostic and treatment facilities, population aging and lifestyle change in these countries.

In the study by Fidler et al. (2017), there was a strong and positive relationship between the overall burden of cancer incidence in the world and the HDI for both sexes, which is mainly due to the increasing adaptation of societies to the behaviors and environment that are typically built in countries. Western is more common (14). Although the current cancer burden in terms of incidence disproportionately affects the most developed countries, countries currently classified as low or medium on the HDI will actually experience the largest proportional increase in future cancer burden (30). Specifically, it has been estimated that low and medium HDI countries will experience a 100% and 81% increase in cancer incidence from 2008 to 2030, respectively (16). Such projected projections emphasize the need for targeted resource-based interventions to reduce the number of future cases, particularly in developing countries. The higher incidence rate in very high HDI populations is consistent with the availability and more common use of prostate-specific antigen testing (31). Because prostate-specific antigen testing has a much greater impact on incidence than mortality, there is less variation in mortality rates worldwide (31). 

In this report, the inequality of YLL and YLD of prostate cancer was observed. However, when the DALY components were evaluated, the contribution of YLL and YLD differed significantly across HDI levels, generally higher total YLL and YLD were observed in high HDI countries, which is consistent with the results obtained by the study of Fidler et al. (14). Among the limitations of this study is that considering that the current study is an ecological study, the most important error in this study is ecological fallacy, and the results of these studies should be interpreted with caution. Also, the limitations reported in GBD studies and the lack of accurate and reliable data for incidence and mortality rates in some countries, especially in countries with a low human development index, can be mentioned.

This review showed how changing levels of human development affect the scale and profile of prostate cancer in Asian countries, and highlighted the disparities in disability and years lost due to premature death from this cancer. It shows specific and cost-effective cancer control strategies that can reduce and minimize the growing gaps in cancer burden between the least and most developed countries in the coming decades. By providing primary prevention methods and conducting epidemiology studies, timely treatment, follow-up of prostate cancer patients, especially for people from less developed countries, an effective step can be taken in reducing the disease burden and improving the health system of countries**.**
